# Supplementary material for: Sensor orientation and other factors which increase the blast overpressure reporting errors
Source: PLoS One. 2020 Oct 8;15(10):e0240262. doi: 10.1371/journal.pone.0240262 (PMC7544144; doi:10.1371/journal.pone.0240262)
Supplement: Attachment — Submitted filename: Response to reviewers.docx [file pone.0240262.s002.docx]

We would like to thank reviewers and the editorial office for the speedy turnaround with the first round of reviews. Second, we would like to extend our gratitude to both reviewers for their time and criticism. We thoroughly revised the manuscript and modified it according to your suggestions. Our answers to specific comments are in blue.

Reviewer #1: References for BOP 2 psi should be supplied.

I do not think we mentioned anywhere in the manuscript the "BOP 2 psi". Possibly 4 psi safety level was questioned (?). If that is the case, in the abstract, we refer to the 4 psi as currently adopted safety threshold. It is a limit accepted within military and law enforcement, and it is based on the eardrum rupture limit. The synopsis is given in the ref. 19 (UFC 3-340-02) on page 11, where human tolerance to blast is presented.

Line 63: Clarify helmet locations here and in Ln113.

RE: Line 63. The configuration described in line 63 is the so-called 'DARPA config', which is a configuration developed by the manufacturer of the Blast Gauge, BlackBox Biometrics (B3). These sensors are labeled as H, S, and C, with the designated locations on the head, shoulder, and chest, respectively. We did not use this configuration in any of the tests described in this manuscript. In our case, we used 4 sensors. They are attached on the surface of the helmet, as presented in figures 3A and 3B.

RE: Line 113. This comment is ambiguous, but I'm going to answer it to the best of my ability. In that paragraph, we described the experiments which compare PCB and B3 sensors. For that purpose, we used a 6" x 6" frame. We didn't use any helmets in these tests, as illustrated in fig. 1 (revised). The location of pressure sensors in the incident and reflected pressure orientation is now clearly identified. Hopefully, that answers the question of the reviewer.

Line 138: Delete building references use biological findings where available.

I am going to assume that this comment refers to line 318, and references in question are 19, 29, and 30:
19. US Department of Defense. Structures to resist the effects of accidental explosions. Washington, 534 D.C.2008 12-05-2008.
2*9. Kinney GF, Graham KJ. Explosive shocks in air: Springer Science & Business Media; 1985.*
30. FEMA-426: Reference Manual to Mitigate Potential Terrorist Attacks against Buildings. Federal 563 Emergency Management Agency Washington, DC; 2003.

There are no biological references that detail the relationship between the incident and reflected pressure like it is done in these references. Moreover, ref. 29 is a widely known book and, as of today (Aug. 5^th^, 2020) was cited 1416 times, which includes many references on biological aspects of the blast. In ref. 29 the relationship between the incident and reflected pressure is devised for ideal gas conditions, while refs. 19 and 30 expand this model in real-world applications. The same correlations which hold for buildings are also valid for a biological specimen as far as the geometry is concerned.

Line 397: Is "medical evacuation necessary? What clinical criteria? This may be force depleting based on ?? P measurement (now it is line 451).

The reviewer is correct that inaccurate pressure measurements can lead to force depletion. The same is true also for averaging the pressure readings from a few sensors. Both effects are demonstrated in our manuscript. We are not claiming that at the present time, there are any medical exclusion criteria, nor there exists the threshold value for the pressure, which would result in removal from duty. On the contrary, our results point out that these parameters should be carefully evaluated, and this analysis will help formulate informed decisions. It is worth to re-iterate that currently there are no standards for occupational overpressure exposure.

General: This paper needs a section on strengths and limitations.

We added a section, which describes the most important strengths and limitations if these are not mentioned in the conclusion section (lines 461-471).

Reviewer #2: The authors present a very important study for the blast community that will help researchers have a better understand blast instrumentation, specifically pressure sensors. This fundamental knowledge is a necessity for all blast experiments and it is a significant flaw in many historical studies. If the basic knowledge of sensor reporting and its data collection are not legitimate, study results cannot be comparable and lead to misunderstanding of results that affect military and civilian policies. This is very important work, however, major revision are needed for better clarity of the study and its results.

• A significant concern is regarding the different sampling rats used for the shock tube testing and the effect it could have on the results. The authors report a large difference in sampling rate between B3 and PCB sensors. If B3 is limited, the PCB sensors could have been sampled at the same lower rate to ensure differences are due to sensors and not sampling rate. This would answer many questions about the data. The authors should repeat the PCB tests with the 100 kHz sampling rate. This may have significant effect on the collection of data. Is the 'lower' peak pressure because of the lack of capturing the actual peak? It is important to find the answer to this question.

The question which is answered here is not whether B3 and PCB sensors should be compared at the same sampling frequencies, but whether B3 sensors can record the pressure waveforms with a high degree of fidelity at their operational sampling frequency. The sampling rate for B3 sensors is 200 kHz (corrected in the revised manuscript), which for a sensor with an adequate response that is sufficient to capture the peak incident and reflected shock waves. It is obvious when the rising part of the pressure waveform is enlarged. We added four insets to figures 1D-1F, that illustrate the differences in the response between B3 and PCB sensors. The pressure increase from the baseline to the peak takes 1-2 microseconds for PCB sensors, while for B3, it takes 50 microseconds, and the pressure reaches only 50-60%% of the pressure recorded by the PCB sensor. Hence, the inadequate sensor response is responsible for reflected pressure undermeasure.

• Understand sensor placement is critical to read the data. Figure 1 should be revised to indicate where each sensor is located so the reader can better identify which sensor is.

The sensor locations are described in the experimental section (line 118): "The incident pressure sensors were mounted at the 90° incident angle, while the reflected pressure sensors were mounted at the 0° incident angle, with respect to the direction of the shockwave propagation (facing the incoming shock wave)."

The sensor array used in the study is presented from an angle in figure 1A, and from the front in figure 1B. The 12 B3 sensors attached at the perimeter are in the incident pressure orientation, whereas 8 B3 and 3 PCB sensors (labeled as 29, 30, and 31, fig. 1B) are in the orientation to measure reflected pressure. We modified figure 1 and incorporated these suggestions, i.e., the reflected pressure sensors are outlined by a white rectangle. Figure 1 legend was also updated.

• Figure 1 legend needs to be revised as states panel 'D' twice instead of 'E'

Corrected.

• How many of each sensor type used was a bit confusing? There is some detail later in the results but should be in the original methods section.

The sensor numbers and their mounting and orientation are specified in the materials and methods section:

Line 118: "Three PCB sensors (model 102B06) were then mounted on the test fixture attached to the frame made of t-slotted framing rails (McMaster-Carr, Robbinsville, NJ), and used as a reference to measure the reflected overpressure (Fig. 1A, B). Next, the B3 Blast Gauges were attached using the elastic mounting band supplied with the sensor. A total of 23 sensors were used to measure the incident overpressure, while 16 sensors were used to evaluate the response in the reflected pressure mode. The incident pressure sensors were mounted at the 90° incident angle, while the reflected pressure sensors were installed at the 0° incident angle, with respect to the direction of the shockwave propagation (facing the incoming shock wave).

Line 158: "The reflection coefficients for B3 sensors used a combination of the incident (12 or 11 sensors) and reflected pressure (2 x 8 sensors), which were repeated ten times at both BOPs.

These details are also included in the figure 1 legend in the revised version of the manuscript.

• The dimensions of the fixture shown in Figure 1 should be provided in the methods. It appears that is would take up a large portion of the shock tube cross-sectional area.

The fixture is 6 x 6 inches, while the cross-section of the shock tube is 28 x 28 inches, which is stated in the original version of the manuscript (line 99: "The 28" x 28" square cross-section shock tube…"). The fixture-to-shock tube cross-sectional area ratio is less than 5%.

The information about the size of the fixture was added in the experimental section of the revised manuscript (line 119).

• Methods are repeated and even more thoroughly explained in the results section – could be concisely stated in the methods, to begin with.

These were removed and replaced with the sensor response results.

• Why were such low pressures used (5 and 10 psi)? To mimic breaching exercises? This could be justified/discussed within the introduction. Currently, only a sentence in the abstract.

The two pressure levels were selected based on typical overpressures in training and are well documented in the existing literature on the subject. The details can be found in Kamimori, et. al, Occupational overpressure exposure of breachers and military personnel. Shock Waves 2017, 27, (6), 837-847. In that paper, there are 11 tables which list overpressure ranges associated with different weapon systems. Pressure ranges reported: <1 psi to 12 psi. Pressure ranges are also reported in more recent papers from our group (refs. 1-6). Considering the amount of information on the subject, it is not worth including in the introduction. We've added a sentence in the introduction to the discussion (line 335): "We performed this evaluation at the two pressure levels, which are within typical range likely to occur in military training environment [2, 3]."

• Duration for all test should be listed. This might help explain the impulse differences. Was one duration time used for all calculations?

There are two distinct terms which should not be confused: 1) shock wave duration, and 2) integration time (interval). The duration for all incident and reflected waves (at 5 and 10 psi BOP) is approx. 4 milliseconds, which is illustrated in figures 1D-1F.

The integration time is different for B3 and PCB sensors: 20 ms and 4 ms. The B3 has the procedure for impulse calculations embedded in the firmware, and the signal is integrated over 20 milliseconds, which is stated in the original manuscript in:

- line 111: "…integration time is 20 ms."

- line 311: "The evaluation of the impulse waveforms revealed that B3 sensors consistently overestimate these by a margin of 11-23%. The consistency of these results likely stems from the algorithm of pressure waveform integration embedded in the firmware, which uses the entire 20 ms of the recorded signal, (i.e., it adds the integral of the baseline noise to the reported value)."

• Why was a different helmet used in field-testing than in lab testing? ACH vs ECH. Similarly, why was a different head form used?

ACH was until recently a standard combat helmet, and it is gradually replaced with modern designs with improved performance. While these two helmets are made of different materials (Kevlar and UHMWPE), their shape is very similar and is not a factor in pressure measurements described in this manuscript.

The headform for shock tube testing is a property of the NJIT lab where it was used for other projects. Headforms for field testing have different requirements because of the nature of these tests: portable, expendable, and their number can be easily increased depending on the testing requirements. From the perspective of pressure measurements on the helmets both types are merely an attachment point for the helmet.

• "The incident peak overpressure is higher at 5 psi, and matches the PCB sensors at 10 psi." Line 193, could use some clarifying – B3 sensor is higher/lower than PCB reference sensors?

Corrected. It reads: "For B3 sensors the incident peak overpressure is higher at 5 psi and matches the PCB sensors at 10 psi."

• Says at line 192 that the impulse values are overestimated, but in line 195 says they are largely underestimated for B3 sensors?

Line 192 is about the impulse (fig. 2B), while line 195 is about reflection coefficient (fig. 2C). No changes.

• Justification for some experimental design aspects is lacking, e.g. why did they select the averaging techniques?

The rationale is the following: if there are four sensors used for pressure monitoring, the available options are to use the BOP and impulse from a single sensor or an average. In the abstract (line 29) a summary of used averaging methods is provided: "The evaluated methods included averaging peak overpressure and impulse of all four sensors, taking the average of the three sensors, or isolating the incident pressure equivalent using two sensors." This is an exhaustive analysis that focuses on imitating the three-sensor setup, the DARPA configuration, which is described in detail in the introduction (lines 59-64). We also included the averages of four and two sensors as a reference, and there are also data from individual sensors. The critical part is the conclusion of the averaging analysis, which can be extrapolated to any similar analysis: "…the inclusion of the reflected pressure sensors leads to an increased peak overpressure and impulse values."

Also (line 272): "The number of sensors, their location, and post-processing algorithms are subject of contention, and focused systematic research is needed to establish the knowledge base necessary to formulate these standards. We focused on three important aspects: performance of currently utilized wearable sensors, their orientation, and evaluation of rudimentary post-processing algorithms."

• Post processing of data is not mentioned and should be described.

No post-processing was necessary on any data included in the manuscript.

• Overall, there are several sentences with unnecessary commas and sentences where commas would have been very helpful to clarify the point. Grammatical proofreading would make this much easier to read.

The manuscript was revised and proofread independently by two native English speakers.
