# Supplementary material for: Sensor orientation and other factors which increase the blast overpressure reporting errors
Source: PLoS One. 2020 Oct 8;15(10):e0240262. doi: 10.1371/journal.pone.0240262 (PMC7544144; doi:10.1371/journal.pone.0240262)
Supplement: Attachment — Submitted filename: Response to reviewers.docx [file pone.0240262.s003.docx]

We would like to thank both reviewers for their time and criticism. We carefully revised the manuscript and modified it according to your suggestions. Our answers to specific comments are below marked in blue.

Reviewer #1: Questions answered but the MS can still be made more clear by clarifying comments Eg beginning with the phrase, "It is true..." What exactly is true?

We carefully edited the manuscript and eliminated the ambiguous parts following the reviewer's suggestion.

Also, the charge used in the open exposures, its distance, suspension needs to be better clarified.

The details of the charge and the distance are included in the revised manuscript. The mounting of the charge is a low-tech solution that utilizes tape and a prop stick. The charge size and mounting methods are routinely used at that breaching training school.

The response to the solid wall references seems inadequate.

This comment is unclear. Please, be more specific to which part of the text you are referring to. The line numbers are there for that purpose. That was addressed in our previous response, so we are going only to add more to what was already said. For the original response, please refer to the answers given in the previous review.

We can only assume that the point of contention is still the description of the relationship between **reflected and incident pressure** (lines 381-383 in the revised manuscript and references 19, 29, and 30). We could not find any references where this relationship was derived for biological materials (which was the reviewer's original comment). The reflected pressure depends on the intensity of the incident pressure and the angle of incidence, and these relationships are presented as a series of plots in references 19, 29, and 30. This problem rests in its entirety in the engineering domain, and it is described briefly in the manuscript to illustrate the difference between reflected and incident pressure. We are not concerned about other aspects of blast wave interaction with structures, including biological specimens.

The section on limitations needs expansion, particularly comparing the open field and tube.

A short description of three factors as potential limitations is included in the revised manuscript (line 456 onwards):

"Moreover, the averaging analysis was done on uncorrected peak overpressure and impulse values, as these values would not be known generally without reference pressure gauges. Applying corrections would likely lead to higher divergence of results, particularly in peak overpressure evaluation. The impulse values in both shock tube and field tests (approx. 17 psi∙ms and 4 ms duration (incident)) are also on the upper end of these experienced in training. An additional study that uses short duration/lower impulse shock waves representative for other training environments would be needed to confirm our findings. Lastly, the intermediate helmet orientation angles, e.g., 45 degrees, might result in different pressure distribution patterns at the four reference points used in this work."
